# Research progress on the mechanism of glycolysis in ovarian cancer

**DOI:** 10.3389/fimmu.2023.1284853

**Published:** 2023-11-28

**Authors:** Chan Li, Fang-Yuan Liu, Ying Shen, Yuan Tian, Feng-Juan Han

**Affiliations:** ^1^ Heilongjiang University of Traditional Chinese Medicine (TCM), Harbin, Heilongjiang, China; ^2^ The First Affiliated Hospital of Heilongjiang University of Traditional Chinese Medicine (TCM), Harbin, Heilongjiang, China; ^3^ Zhejiang University of Chinese Medicine, Hangzhou, Zhejiang, China

**Keywords:** ovarian cancer, glycolysis, hexokinase, phosphofructokinase, pyruvate kinase

## Abstract

Glycolysis is the preferred energy metabolism pathway in cancer cells even when the oxygen content is sufficient. Through glycolysis, cancer cells convert glucose into pyruvic acid and then lactate to rapidly produce energy and promote cancer progression. Changes in glycolysis activity play a crucial role in the biosynthesis and energy requirements of cancer cells needed to maintain growth and metastasis. This review focuses on ovarian cancer and the significance of key rate-limiting enzymes (hexokinase, phosphofructokinase, and pyruvate kinase, related signaling pathways (PI3K-AKT, Wnt, MAPK, AMPK), transcription regulators (HIF-1a), and non-coding RNA in the glycolytic pathway. Understanding the relationship between glycolysis and these different mechanisms may provide new opportunities for the future treatment of ovarian cancer.

## 1 Introduction

Ovarian cancer (OC) is a common malignancy in gynecology, with approximately 310,000 women diagnosed and 200,000 deaths each year worldwide (1). The insidious nature of the early stage of the disease means that most patients have an advanced form of the disease at the time of diagnosis, and consequently, the disease has an extremely high mortality rate. Risk factors for OC include a family history of ovarian or breast cancer, age, endometriosis, obesity, early menarche, and menopausal hormone therapy (2). The main treatment methods for OC include surgery, radiotherapy, chemotherapy, and immunotherapy, but more than half of patients will have adverse outcomes such as recurrence, metastasis, and chemotherapy resistance.

Metabolic reprogramming is a distinguishing hallmark of cancer, and glycolysis is a central factor in cancer progression (3). In normal cells, glucose maintains a state of homeostasis. In the 1920s, Warburg proposed that cancer cells preferentially rely on the conversion of glucose to pyruvate, and then to lactate, to produce large amounts of energy, even if the oxygen content is normal (4). This transformation of glucose utilization from oxidative phosphorylation (OXPHOS) is called glycolysis and is predominantly characterized by increased rates of glucose uptake and lactate production. Glycolysis produces ATP in quantities smaller than those of OXPHOS but may be up to 100 times more efficient, which, in turn, promotes higher glycolytic activity (5). This low-yield and high-rate glycolysis mode not only generates the ATP required, but also produces more substances such as nicotinamide adenine dinucleotide (NADPH), which are required for glycogen, protein, and nucleotide synthesis, thereby allowing cells to quickly adapt to energy- and nutrient-deficient microenvironments.

## 2 OC tumor microenvironment

The tumor microenvironment (TME) is ecologically complex because it contains various growth factors, chemokines, cytokines, and angiogenic factors, and it plays a crucial role in cancer. In the TME, cancer cells require increased glycolytic activity to compensate for the large amounts of energy and nutrients that their abnormal metabolism requires.

**Figure 1:**
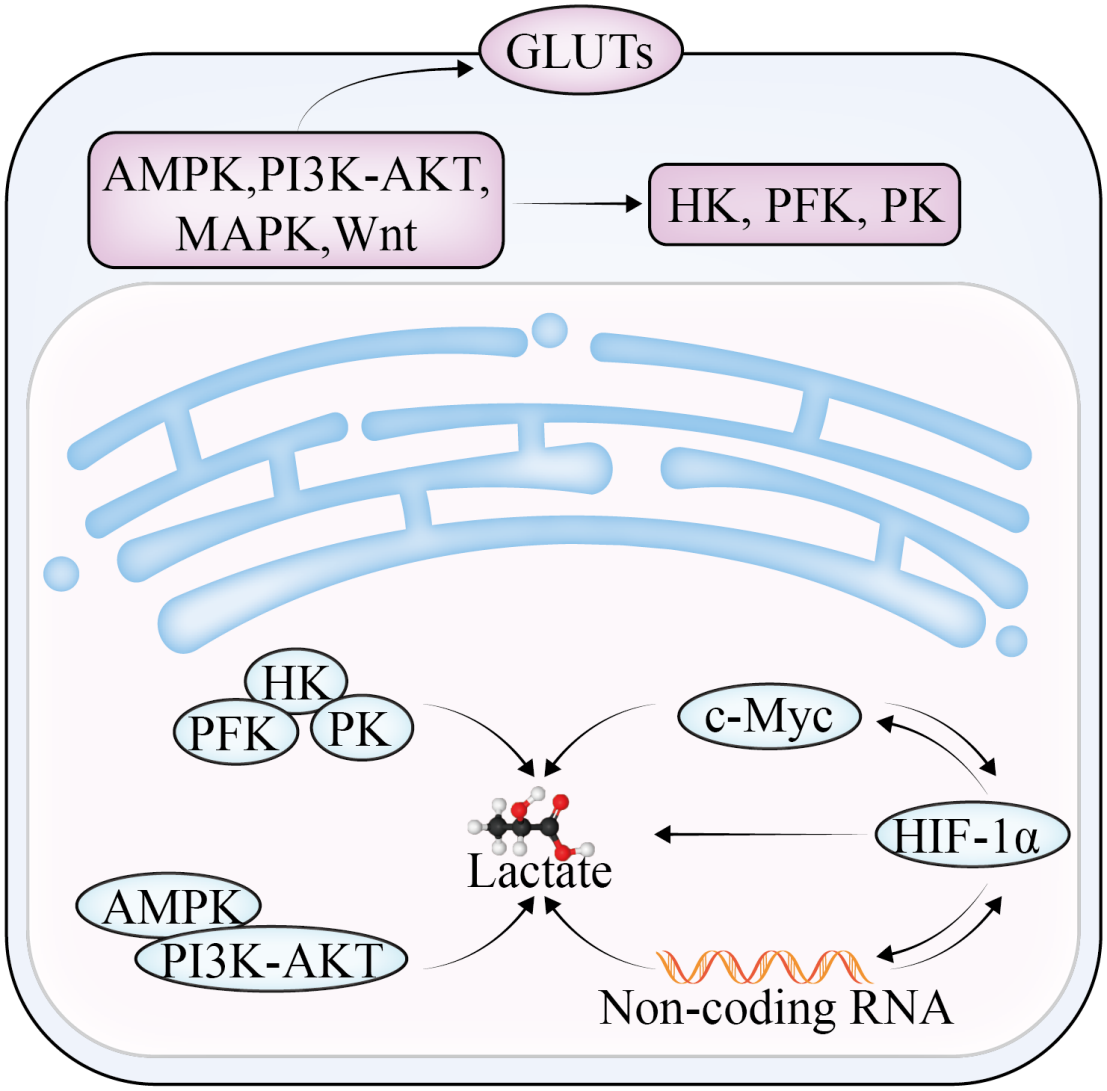
The regulatory mechanism of lactate in glycolysis in OC.

Lactate, which is the end product of glycolysis, is an immunomodulatory compound that can be absorbed by endothelial cells (6) and related fibroblasts, triggering the production of multiple cytokines and vascular endothelial growth factor and promoting OC invasion, metastasis, and drug resistance (**Figure 1**). The overproduction of lactic acid leads to acidification of the TME, forming an immunosuppressive microenvironment, which is a key mechanism of cellular immune escape. Lactate is also the main energy source of mitochondrial metabolism, which is involved in inhibiting the proliferation of immune effector cells and inducing immune cell dedifferentiation (7, 8). In addition, lactate has been reported to not only reduce the activity of natural killer (NK) cells and natural killer T (NKT) cells (9), but also promote the proliferation of T lymphocytes and accelerate the establishment of immunosuppressive microenvironment. Furthermore, lactate can affect the growth and apoptosis of cancer cells by regulating the function of tumor-associated macrophages (TAM). TAM can be divided into two subtypes—M1 and M2—according to their activated phenotype. The M1 TAM are proinflammatory macrophages that are dependent on glycolysis and inhibit the proliferation of cancer cells; the M2 type are anti-inflammatory macrophages that rely on oxidative phosphorylation to promote the proliferation and metastasis of cancer cells. Lactate was found to induce TAM polarization to the M2 phenotype and promote tumor growth in the TME (10).

Numerous studies have shown that glycolysis (11) can participate in multiple stages of OC development by promoting cancer cell proliferation, invasion, metastasis, and chemoresistance, and thus influencing prognosis. Liu et al. (12) found that enhanced glycolytic activity was linked to poor prognosis in OC and a low survival rate. Inhibitors of phosphoglycerate kinase (PGK1), which can serve as an independent risk factor for OC survival and prognosis (13), reduce epithelial–mesenchymal transition (EMT) and reverse the glycolytic effect, which, in turn, reduces the capacity of OC cells to migrate and invade. Phosphoglycerate mutase (PGAM1) can mediate the synthesis of lactate and pyruvate and participate in regulating the resistance of OC cells to the chemotherapy drug paclitaxel (14). In addition, Bi et al. (15) finding in constructing a prognostic model of OC associated with glycolysis that gene characteristics are related to the TME, especially immune cell infiltration and expression of immune-related genes, which can be used as a potential therapeutic target for OC. Overexpression of mitochondrial elongation factor 2 (MIEF2) can promote the transformation of OC from OXPHOS to glycolysis (16), whereas inhibiting the expression of MIEF2 may induce cell cycle arrest and apoptosis. In addition, metabolic intermediates produced during glycolysis can be used for macromolecular biosynthesis in cancer progression. It was reported (17) that downregulation of glucose-6-phosphate isomerase, fructose-bisphosphate aldolase, lactate dehydrogenase (LDH), and phosphoglycerate kinase (PGK1) expression was directly correlated with drug resistance in the established OC cisplatin-resistant cell line A2780-DR, and overexpression of Ras-related proteins enhanced the sensitivity of these cells to cisplatin therapy. The long-chain coding RNA NRCP (18) leads to the overexpression of downstream glucose-6-phosphate isomerase, which increases sensitivity to cisplatin when targeting siRNAs with NRCP using DOPC nanoliposomes. In the OC A2780 cell line, sphingosine kinase-1 (SK1) is a crucial enzyme in a metabolic pathway that supports macromolecular biosynthesis (19). This finding was demonstrated by upregulated expression of the proton/monocarboxylic acid transporter MCT1 and GLUT3, increased lactate production, and activation of the AKT pathway.

Recently, the role of glycolysis in OC has received much attention, with glycolysis being a marker of OC progression and a potential therapeutic target. Glycolysis inhibitors, traditional Chinese medicine (TCM) ingredients, and chemotherapy drugs have been reported to affect the incidence and progression of OC by knocking down or reversing glycolysis activity. However, there are no systematic studies and discussions on how glycolysis regulates and influences the progression and treatment of OC. Therefore, we summarize the roles of several key enzymes, signaling pathways, cellular transcription factors, and non-coding RNAs (ncRNAs) involved in glycolysis in OC to supply novel insights for the treatment of this malignancy.

## 3 Key enzymes of glycolysis as potential targets for OC

**Figure 2:**
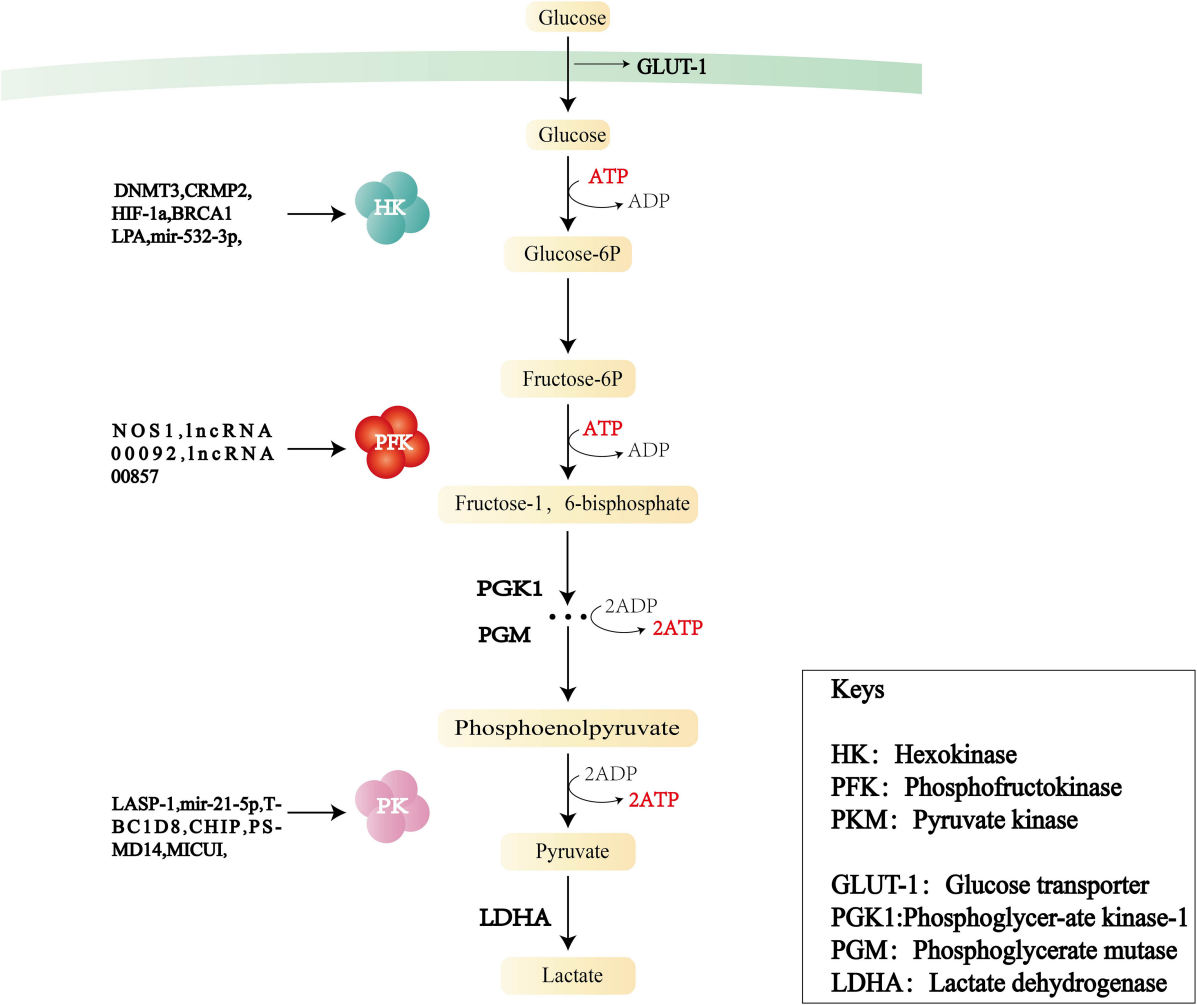
Flowchart of glycolysis and the role of three key enzymes—hexokinase (HK), phosphofructokinase (PFK), and pyruvate kinase (PKM)—in OC.

During the progression of cancer, the increase of glycolysis enzyme activity is proportional to the rate of glycolysis. The rate-limiting enzymes of the glycolysis pathway are HK, PFK, and PKM (**Figure 2**). Changes in the activities of these enzymes play a key role in the pathogenesis of OC consequently, the enzymes can be used as prognostic indicators and therapeutic targets of OC.

### 3.1 Hexokinase

The first rate-limiting enzyme in the glycolytic pathway is HK, which converts glucose into glucose 6-phosphate (G-6-P) through phosphorylation and is a key molecule regulating cell energy metabolism.HK consists of five major isoforms: HK1, HK2, HK3, HK4, and HKDC1. HK1 is mainly found in the brain. HK2 predominantly exists in insulin-sensitive tissues and tumor cells, and its functions include metabolic rewiring of glycolysis, regulation of autophagy, and shielding from cell death stimulation. HK3 is mainly distributed in the bone marrow, lungs, and spleen. HK4 can regulate insulin secretion, glucose uptake, and glycogen synthesis and decomposition in the liver. HKDC1, a recently discovered isozyme, is adjacent to the HK1 gene.

In OC, the increase in HK activity is related to the overexpression of HK2, especially in ascites (20) and metastatic foci. HK2 can control lactate production and promote OC metastasis through the expression of MMP9/NANOG/SOX9 mediated by the FAK/ERK1/2 signaling pathway. The increased activity of HK2 can also participate in angiogenesis. HK2 (21) is influenced by several signaling pathways and transcription factors, including PI3K/AKT, FAK/ERK1/2, HIF-1, RAS, and STAT2. Zhang et al. (22) found that DNMT3A can promote glucose consumption and lactate production by upregulating the expression of HK2 and PKM2. CRMP2 (23) can affect angiogenesis by activating the expression of HIF-1 and HK2. Lysophosphatidic acid, a blood-derived lipid medium, is abundant in the ascites of patients with OC, and was found to selectively upregulate HK2 (24), subsequently inducing cell proliferation and enhancing the glycolysis rate and lactate production. HK2 levels are also regulated by microRNA (miRNA) after transcription. Tuo et al. (25) reported that miR-532-3p inhibited OC cell proliferation and invasion through the GPNMB/HIF-1α/HK2 axis. In addition, BRCA1 (26) deficiency can increase glycolytic activity by inducing the transcription factors MYC and STAT3 to activate HK2 expression. Meanwhile, BRCA1 deficiency decreases fatty acid oxidation, enhances triphosphopyridine nucleotide generation, and participates in the upregulation of OXPHOS via the pentose phosphate pathway. This upregulation of HK2 induced by BRCA1 deficiency could be partially reversed by aspirin.

In summary, HK2 is closely related to the energy metabolism, proliferation and invasion, lactate production, and angiogenesis of OC cells, and may be an OC prognostic indicator and treatment target.

### 3.2 Phosphofructokinase

Fructose-6-phosphate kinase 1 (PFK1) is the second rate-limiting enzyme of the glycolytic pathway. It catalyzes the conversion of fructose 6-phosphate to fructose-1,6-fructose diphosphate. This irreversible step determines the rate and level of glycolysis and is the critical regulation point in the glycolysis process. PFK1 acts in the form of a homomeric or heteromorphic tetramer, including catalytic sites and regulatory sites of allosteric effectors. When the concentration of ATP is high, it can bind to the regulatory site, change the conformation of the enzyme, and prevent ATP from binding to the catalytic site, resulting in a decrease in enzyme activity. PFK2 is a bifunctional protein: in the non-phosphorylated state, it has the function of a kinase, phosphorylating fructose 6-phosphate(f-6p) to produce fructose-2,6-bisphosphate (F-2,6-BP), whereas in the phosphorylated state, it transforms into a phosphatase and catalyzes the conversion of 2-6 diphosphate fructose to 6-phosphate fructose. In addition, F-2,6-BP—a key activator of PFK1–is produced by PFKB2 (27); it is a product of f-6p catalyzed by PFKB3 (28), participates in the glycolytic process, and is also regulated and maintained by the dual action of phosphorylation and dephosphorylation by PFKB4.

The PFK family and its metabolites are instrumental in the regulation of glycolysis. High PFK activity is associated with poor metastasis or poorer survival. Jiang (29) et al. demonstrated that PFKB3 was associated with advanced/graded OC and adverse outcomes in experiments conducted both *in vivo* and *in vitro*. PFKM (muscle type) is one of the three isoforms of PFK expressed in most mammals. Endogenous nitride NOS1 (30) has been confirmed to engage in OC metabolic reprogramming by inducing the s-nitrosylation of PFKM at Cys351 to stabilize the tetramerization of PFKM, thereby resisting the negative feedback of downstream metabolic intermediates, leading to an increase in glycolysis and the tricarboxylic acid cycle. Long non-coding RNA (LINC RNA)00092 (31) serves as a key node for cancer-associated fibroblast (CAF)-mediated metastasis. Further studies revealed that CXCL14, a pro-mediator secreted in CAF, induced LINC RNA00092 to bind to PFKFB2 to promote OC metastasis. Moreover, the PFK family has an impact on OC chemoresistance. The activity of PFKFB3 is positively correlated with OC chemoresistance and lipid droplet biogenesis (32), and its inhibitor, PFK-158, targets glycolytic and adipogenic pathways, making chemoresistant cells sensitive to drug-induced cytotoxicity. Yap is a transcriptional activator encoded by paralogous genes and regulated by the Hippo pathway. The lncRNA 00857 can upregulate YAP1 in the Hippo signaling pathway by competitively binding miR-486-5p, thereby enhancing glycolytic activity and promoting the proliferation and migration of OC cells (33). Research (34) has shown that PFK1 and F-1,6-BP can promote positive feedback loops in the phosphatidylinositol 3-kinase (PI3K) and/or YAP/TAZ signaling pathways. Targeting PFK1 and its product F-1,6-BP can improve the clinical efficacy of PI3K and/or YAP/TAZ inhibitors in practice.

### 3.3 Pyruvate kinase

PK is the final rate-limiting enzyme of the glycolytic pathway and catalyzes the conversion of phosphoenolpyruvate to pyruvate with concomitant production of ATP. PK comprises four isoforms: L, R, M1, and M2. PKM2 is the major isoform in proliferating cells and plays a key role in the direct regulation of gene expression and pre-cyclic cellular expression. PKM2 exists in tetrameric and dimeric forms. The tetramer has high catalytic activity and can rapidly convert phosphoenolpyruvate to pyruvate, increasing glycolytic yield and generating more ATP. The dimer has low catalytic activity and can be translocated into the nucleus, acting as a coactivator of HIF-1, NF-κB, STAT3, and PISK-AKT, and promoting the transcription of target genes, positive feedback regulation of glycolysis, and angiogenesis. The ratio between the tetrameric and dimeric forms determines PKM2 activity and the glycolysis flux of catabolism or anabolism. In addition, PKM2 mutants exist mainly as dimers, and their overexpression promotes cell proliferation.

PK affects tumor cell growth, invasion, and angiogenesis, and could be a potential therapeutic target for OC. Evidence suggests (35) that disruption of pyruvate dehydrogenase (PDH) phosphorylation to PDK (PDK–PDH axis) may affect OC progression and chemotherapy resistance. MICU1 regulates mitochondrial free Ca^2+^, and its overexpression is a feature of many cancers. Silencing MICU1 (36) stimulates PDK to activate PDH, reduces lactate production, and increases cisplatin efficacy. When LIM and SH3 protein 1 (LASP-1) (37) is downregulated, it may affect zyxin proteins through the upregulation of PK, inducing G2 phase accumulation in SKOV3 cells (a human OC cell line) and thereby reducing tumor growth and invasion. Pyruvate dehydrogenase kinase 1 (PDK1) (38) blocking the trigger factor is a symbol of cancer cell metabolic changes *in vitro*. Furthermore, silencing PDK1 can reduce angiogenesis and increase tumor cell necrosis in OC316 and OVCAR3 tumor models. In addition, PDHA1 is a carrier gene for an important subunit of PDK1. Exosomal miR-21-5p (39) promotes cell viability and glycolysis and inhibits the chemical sensitivity of SKOV3 progenitor cells by inhibiting PDHA1. Follicle-stimulating hormone has been shown to regulate cellular metabolism in patients with OC, and further studies (40) found that follicle-stimulating hormone increased PKM2 expression in cells by promoting anabolism, which enhanced OC proliferation. CHIP (41), which is an E3 ligase that mediates PKM2 degradation, downregulates PKM2 by promoting its ubiquitination and degradation. Overexpression of CHIP reduces the production of pyruvate and lactate, and thus reduces glycolysis. CHIP can also affect glycolysis by influencing PKM2 nuclear translocation. TBC1D8 (42) reduces PK activity by binding to PKM2 and hindering PKM2 tetramerization while promoting PKM2 nuclear translocation, thereby inducing the expression of genes involved in glucose metabolism and the cell cycle. PSMD14 (43) downregulates PKM2 by decreasing ubiquitylation of the K63 connection on PKM2. This action downregulates the ratio of PKM2 tetramers to dimers and monomers to reduce PK activity while promoting nuclear translocation of PKM2 to enhance transcription of downstream oncogenes, which, in turn, stimulates malignant progression of OC. Moreover, the PSMD14 inhibitor O-phenanthroline inhibits the colony formation and growth of OC cells in a concentration-dependent manner.

## 4 Interplay of signaling pathway and OC

**Figure 3:**
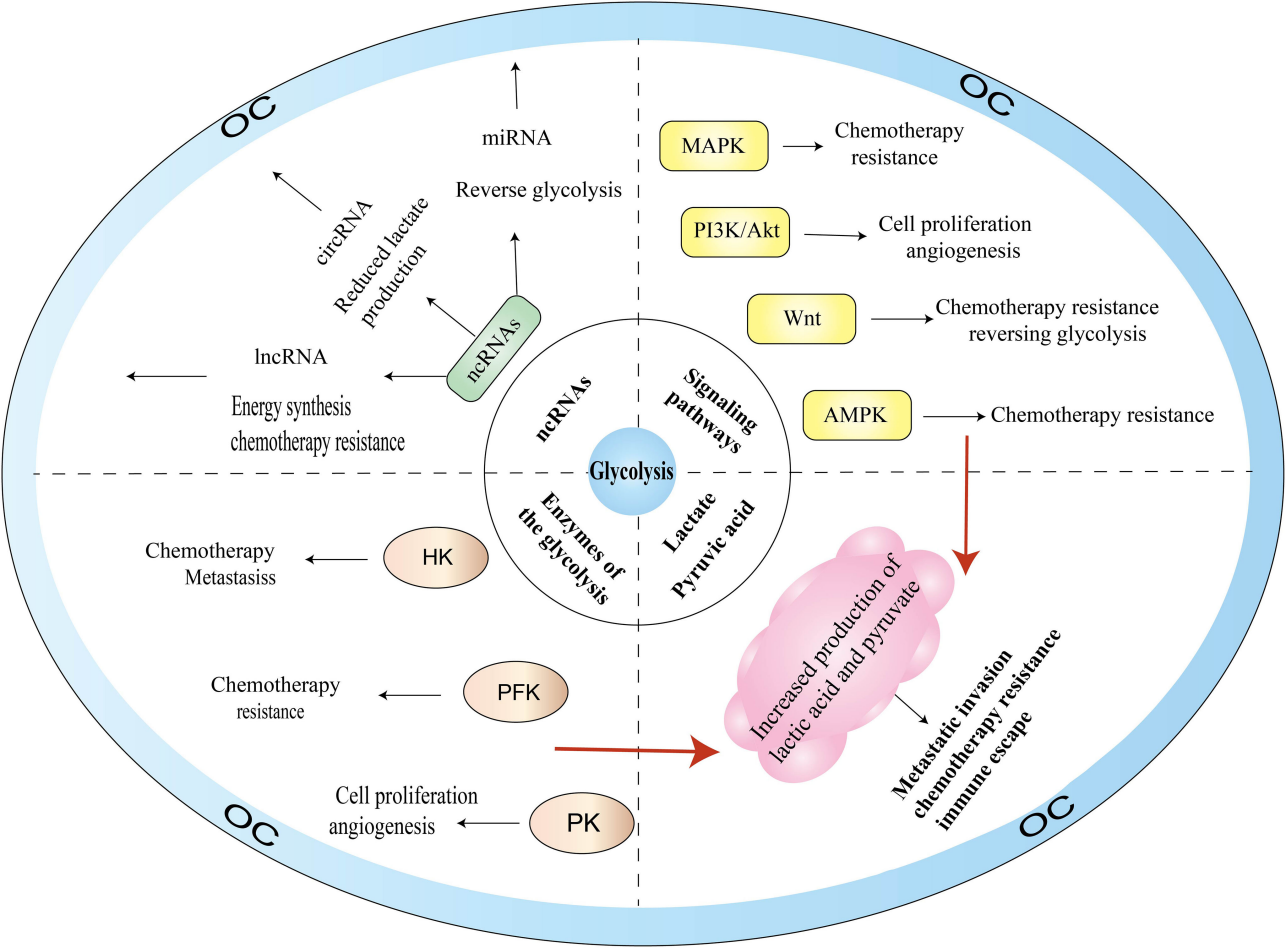
Glycolysis-related pathogenesis of OC.

With the deepening of research on the pathogenesis of cancer at the molecular level, glycolysis is considered to be the main metabolic phenotype of cancer. In this section, we summarize the research on signaling pathways involved in glycolysis and OC, including PI3K/AKT, MAPK, Wnt, and AMPK signaling pathways (**Figure 3**).

### 4.1 AMPK

AMPK is a highly conserved serine/threonine kinase that regulates cellular homeostasis by modulating the ratio of ATP to ADP and plays a pivotal role in the regulation of cellular energy homeostasis, including glucose, protein, and lipid metabolism and autophagy. There are numerous AMPK-related proteins, including salt-inducible kinase (SIK1), QIK (also known as SIK2), QSK (also known as SIK3 kinase), and maternal embryonic leucine zipper kinase proteins. AMPK proteins participate in various reproductive physiological functions, including follicle maturation, menopause, embryogenesis, and oocyte maturation. As a cellular energy receptor, AMPK responds to low levels of ATP and, upon its activation, positively regulates signaling pathways that replenish cellular ATP supply. Glycolytic activity is also associated with the energy state of cells. When the intracellular ATP level is high, HK, PFK, and PKM are inhibited by excess glucose 6-phosphate, citrate, and ATP, respectively, which reduces glycolysis activity; conversely, when ATP is insufficient in the body, glycolysis can be activated through ADP to remove the inhibition of ATP on PFK-1, F-1,6-BP to activate PK, as well as on F-2,6-BP to activate PFK-1.

AMPK dysfunction has a critical role in growth, proliferation, metastasis, and invasion in OC. Gao et al. (44) reported that SIK2 not only promotes mitochondrial fission and inhibits mitochondrial OXPHOS by phosphorylating the serine 616 (Ser616) site of Drp1 but also upregulates HIF-1 by activating the PI3K/AKT signaling pathway. SIK2 can directly activate the transcription of major genes of glycolysis and promote glycolysis. In addition, transient receptor potential 7 (TRPM7) may be a potential interventional therapy target for OC (45). When TRPM7 is silenced, ubiquitination degradation of HIF-1α and AMPK activation are enhanced in OC, which results in the conversion of glycolysis to OXPHOS and consequently inhibits OC proliferation. For OC treatment, the herbal extract resveratrol upregulates AMPK expression, and reduces mTOR expression and activation to inhibit glycolysis in OC cells. AMPK activators (46) not only prevent cancer progression and metastasis but also have a significant effect in improving the efficacy of cisplatin in OC.

### 4.2 PI3K-AKT

PI3K is a dimerized object complex consisting of catalytic and regulatory subunits and includes three major types of lipid kinases—class I (IA and IB), class II, and class III—plus a group of distant relatives that are sometimes referred to as class IV. The level of glycolysis can be affected by oncogenes or tumor suppressor genes directly or indirectly, regulating gene expression and enzyme activity. AKT, as a proto-oncogene, is a serine/threonine protein kinase that can be directly activated by PI3K and inhibits or enhances the activity of target proteins through phosphorylation. Thus, AKT regulates the downstream pathways of mTOR, VEGF, MAPK, and glycolysis, and plays an essential part in the proliferation and survival of the cells, transcription, and protein synthesis. PI3K-AKT signaling (47) is mediated by serine or threonine phosphorylation of a series of downstream substrates and affects cell proliferation and apoptosis, glucose homeostasis, angiogenesis, and invasive metastasis.

The PI3K-AKT signaling pathway regulates OC glycolysis through various mechanisms. First, the PI3K-AKT pathway can regulate the activity and expression of some glycolytic enzymes such as HK2, PFK, and PK. Jia et al. (48) found that the oncogene ACTL6A not only mediated glucose utilization, lactate generation, and pyruvate levels by regulating PGK1, but was also upregulated by follicle-stimulating hormone through the PI3K/AKT pathway, which promoted OC glycolysis. YWHAZ (also known as 14-3-3ζ) (49) affects the glucose uptake rate of OC by downregulating PI3K/AKT phosphorylation. Second, the regulation of glycolytic enzyme expression can be mediated by PI3K-AKT through its modulation of AMPK and HIF expression. Creatine kinase B is a cell membrane isoform of creatine kinase (50). Knockdown of creatine kinase B induces cell cycle G2 arrest by enhancing p21 expression and influencing the pathways of PI3K/AKT and AMPK. These actions decrease the generation of lactate and the consumption of glucose, enhance the generation of reactive oxygen species (ROS) and the consumption of oxygen, and thus inhibit OC cell growth and induce apoptosis, making the OC cells more sensitive to chemotherapeutic drugs. Third, PI3K-AKT activates downstream regulators such as mTOR and VEGF, thereby promoting glycolysis and angiogenesis in OC. AKT is one of the downstream targets of epidermal growth factor receptor, which is overexpressed in approximately 70% of OC cases. Epidermal growth factor receptor is closely linked to metastasis, angiogenesis, and pro-apoptotic and pro-survival signaling levels. FBN1 (51) activates its downstream AKT pathway by mediating the phosphorylation of vascular endothelial growth factor receptor 2 (VEGFR2) and induces the phosphorylation of STAT2 involved in glycolysis and angiogenesis, leading to OC cell chemotherapy tolerance. In addition, FBN1 treatment combined with apatinib enhances OC chemosensitivity.

### 4.3 MAPK

The MAPK signaling pathways are critical for cell proliferation, differentiation, apoptosis, and stress response under normal and pathological conditions. MAPKs consist of four subfamilies—ERK, p38, JNK, and BMK1—and are evolutionarily conserved serine-threonine kinases. Currently, the most widely studied pathway in the MAPK family is the Ras-Raf-MEK-ERK signaling pathway. Ras activation can produce effects with several downstream proteins, including AF6, PI3K, PLCϵ, and Raf. For example, Ras-GTP causes RAF protein to be phosphorylated, and the activated RAF kinase binds to the downstream MEK1/2 and subsequently activates ERK1/2. Activated ERK1/2 induces the expression of genes related to the cell cycle and proliferation and phosphorylates various kinases for OC cell proliferation and adhesion.

ERK1/2 is the only downstream target of MEK kinase, and ERK inhibitors can effectively block the Ras-Raf-MEK-ERK signaling pathway while effectively reversing chemotherapy resistance caused by upstream BRAF and MEK mutations. Sirt6 (52), which is an important oncogenic factor, was found to regulate the ERK1/2-driven phosphorylation of Drp1at Ser616, regulating mitochondrial function, cell invasion, cell proliferation, and glycolysis in OC cells. In addition, the lncRNA AB073614, which is correlated with poor survival in patients with OC, may exert oncogenic effects on OC cells by modulating ERK1/2-mediated signaling pathways (53). Snail (54) is a key inducer of OC EMT and was found to enhance OC sensitivity to chemotherapy by inhibiting phosphorylated ERK and thus inhibiting the glycolytic pathway. Collectively, these findings provide further support for the glycolytic therapy of OC, wherein targeting MAPKs may become a therapeutic strategy.

### 4.4 Wnt

The Wnt signaling pathways are involved in cell growth, development, proliferation, stem cell renewal, angiogenesis, chemotherapy resistance, and immune escape, and are among the critical pathways for promoting OC progression. Wnt/β-catenin is the classical Wnt signaling pathway and consists of the transmembrane receptors of the Frizzled family, CK1, GSK3, and Axin, and the TCF/LEF family of transcription factors. This pathway flips β-catenin intracellularly, and when the cell is not stimulated by Wnt signaling, the cytoplasmic casein kinase CK1a, the glycogen synthase kinase GSK3β, the scaffolding protein Axin, and the adenomatous polyposis coli protein form a destructive complex, with β-catenin at serine residues 45, 33, and 37 (Ser45, Ser33, and Ser37) first by GSK3β phosphorylation and then by CK1a. β-Catenin is eventually degraded by ubiquitination modification via E3 ubiquitin ligase, preventing the nuclear translocation of Wnt/β-catenin.

The mechanism of Wnt signaling to regulate glycolysis in OC can be summarized as follows. First, Wnt regulates the transcription of multiple glycolytic enzymes, including lactate dehydrogenase A (LDHA), pyruvate carboxylase, HK2, PFKB3, and PKM. The lncRNA HOXB-AS3, which regulates glycolysis by downregulating miR-378a-3p targeting LDHA, also activates the Wnt/β-catenin pathway and enhances EMT, decreasing OC cell proliferation and enhancing cisplatin sensitivity (55). TNKS (56), an oncogenic regulator of OC, upregulates pyruvate carboxylase to promote aerobic glycolysis by activating Wnt/β-catenin/snail signaling. The inhibition of TNKS activity not only blocks the cell cycle and induces apoptosis, but also enhances the drug sensitivity of OC. Second, Wnt influences OC progression and therapeutic resistance by regulating the multifunctional differentiation and regeneration of cancer stem cells. Cancer stem cells are a class of tumor cells with self-renewal capacity and are considered to be a major source of drug resistance, cancer recurrence, and metastasis. The miR-1180 released by bone marrow stromal stem cells (57) enhances chemoresistance in OC by activating the downstream components of the Wnt signaling pathway (i.e., Wnt5a and β-linked proteins) and upregulating glycolysis levels. Third, Wnt affects the TME and EMT to promote tumor development. Intraperitoneal (58) delivery of 188Re liposome therapy has been reported to block EMT and reactivate p53 function, shifting the metabolic state from glycolysis to OXPHOS and controlling tumor cell growth.

## 5 Transcription factors and OC

### 5.1 c-Myc

Transcription factors play a key regulatory role in OC. Among them, c-Myc is involved in the regulation of cell proliferation, the cell cycle and metabolic reprogramming, and is a vital oncogene in glycolysis in OC. Many studies have shown that overexpression of c-Myc is significantly associated with poor prognosis in patients. c-Myc is a helix-loop-helix leucine zipper transcription factor that closely links cell metabolism with the occurrence and development of tumors. Under normal oxygen conditions, c-Myc (59) can directly promote glycolysis activity through LDHA. c-Myc can also promote the expression of Glut1, PKM, and HK2, and promote glycolytic activity. Furthermore, c-Myc can synergistically act with HIF-1 on PDK1 to promote the conversion of glucose to lactate. It has been reported that fructose-1,6-bisphosphatase 1 (FBP1) is an important target gene of c-Myc, and knockdown of FBP1 significantly reverses the low expression of c-Myc, increases glucose consumption and lactate production, and promotes OC progression and cisplatin resistance (60). The serine/threonine kinase PIM1 (61) activates the expression of PGK1 and LDHA by mediating c-Myc phosphorylation, which regulates glycolysis, and the PIM1 inhibitor SMI4a induces chemical sensitization of cisplatin. In addition, c-Myc is closely linked to other transcription factors and signaling pathways, such as HIF-1a, the Wnt signaling pathway, and the ERK signaling pathway. For example, HK2 (62) promotes OC cell proliferation and tumor formation through Wnt/β-catenin-mediated upregulation of c-Myc. MiR-1180 (57) also can upregulate glycolysis through the activation of Wnt/β-catenin and c-Myc, thus enhancing the chemotherapy resistance of OC cells. Based on the key role of c-Myc and glycolytic interactions in OC tumorigenesis, c-Myc may be a promising therapeutic target.

### 5.2 HIF-1a

Hypoxia (63) is a prominent feature of the TME and is positively correlated with tumor cell growth and metastasis, angiogenesis, and chemoresistance. HIF is a transcription factor in the nucleus that promotes cellular adaptation to hypoxic environments, and includes HIF-1a, HIF-2a, and HIF-3a. HIF-1a is overexpressed in OC and holds a prominent position in multiple pathways of OC development. HIF-1a can regulate the transcription of various genes, including erythropoietin, Glut1, HK2, PFK1, PKM, and VEGF, which promotes the rate of glucose uptake or lactate production. In addition, intracellular ATP is a key determinant of chemoresistance. Under hypoxic conditions, glycolysis is the main energy production pathway, and sensitizing cancer cells requires depletion of intracellular ATP by inhibiting glycolysis.

HIF-1a is frequently upregulated in OC, and its inhibition is an effective strategy for treating OC, including inhibiting glycolysis and improving platinum resistance. After interacting with CAFs, OC cells can mobilize glycogen as an energy source, playing a crucial role in cancer progression, metastasis, and treatment resistance (64). Lysophosphatidic acid, which is a lipid growth factor and GPCR ligand, was found to stimulate aerobic glycolysis in cancer cells by inducing a pseudo-hypoxic response via upregulation of HIF1α levels (65); this response is involved in the transition of normal fibroblasts to a CAF phenotype. Another study found that lysophosphatidic acid (66) stimulates a pseudo-hypoxic response through Rac-mediated activation of triphosphopyridine nucleotide oxidase and ROS production, which activates HIF-1α, induces Glut1 and HKII expression, and affects the metabolic reprogramming of OC cells. Overexpression of the lncRNA GHET1 (67) promotes glucose uptake and lactate production in OC by blocking HIF-1α degradation and upregulating the protein level of HIF-1α, ultimately leading to proliferation and colony formation. Conversely, inhibiting GHET1 induces G1 cell cycle arrest and apoptosis. In addition, lactate has been shown to stabilize HIF1α in the stroma, leading to increased production of proangiogenic cytokines. Ai et al. (68) showed that degradation of HIF-1 converts aerobic glycolysis to mitochondrial OXPHOS in platinum-resistant cells, resulting in overproduction of ROS and induction of apoptosis. Cryptotanshinone (69) inhibits glucose uptake and lactate production by inhibiting the STAT3/SIRT3/HIF-1a signaling pathway. Ginsenoside (70) inhibits the HIF-1α-stimulated Warburg effect in OC via the regeneration of DNMT3A-mediated upregulation of miR-519a-5p by DNA methylation. The HIF-1α inhibitor EF24 (71) blocks the response to glucose uptake, the rate of glycolysis, and lactic acid production by downregulating GLUT-1 expression and thereby reversing the OC glycolytic effects. Therefore, targeting HIF-1α/cisplatin in combination with HIF-1α inhibitors or degraders could provide a new opportunity for the treatment of individuals with platinum-resistant cancers.

### 5.3 Non-coding RNA

Non-coding RNA (ncRNA) accounts for over 90% of the human genome, from which it is transcribed as protein-free RNA. NcRNAs engage in a variety of biological processes including development, proliferation, post-transcriptional modification, apoptosis, and cell metabolism. ncRNAs can be divided into lncRNA, miRNA, and circular RNA (circ RNA). LncRNAs, which are more than 200 nucleotides in length, have a broad variety of functions, such as transcriptional regulation and modulation of gene expression, and are considered potential targets for diagnosis and therapy. In addition, lncRNAs participate in the glycolytic pathway through HKII, PFK, PKM, Glut1, and c-Myc and associated signaling pathways to regulate energy metabolism in tumors. MiRNAs are approximately 22 to 23 nucleotides in length, and more than 60% of coding genes are potential regulatory targets for miRNAs. In terms of glycolysis, miRNAs can target key enzymes in glucose catabolism and ATP production in beta cells (72). CircRNAs are single-stranded circular RNA molecules involved in regulating transcription or influencing gene expression.

ncRNAs can regulate OC glycolysis through the glycolytic enzymes or various signaling pathways (**Table 1**). Overexpression or reduced expression of ncRNA molecules can affect OC energy synthesis, translocation, and the progression of drug resistance by regulating glycolytic activity. In addition, lncRNAs act as “sponges” that can competitively bind to miRNAs and reduce the inhibition of target genes by miRNAs. The lncRNA NEAT1 (73) can directly target BZW1 through the target mir-4500 to upregulate the glycolytic activity of OC cells *in vivo*, which can contribute to the proliferation and colony formation of OC. The lncRNA oiP5-aS1 (74) can upregulate CCNG1 by targeting mir-128 and plays a tumorigenic role in the development of OC. Decreased expression of lncRNA 00504 (75) simultaneously reduces the expression of PKM2, HK2, and PDK1, and alters aerobic glycolysis in OC. The circ_0025033 (76) inhibits glucose consumption and lactate generation by targeting miR-184 to upregulate LSM4 expression. Lin and colleagues found that the circRNA ITCH (77) regulates the level of CDH1 protein by targeting miR-106a to inhibit proliferation, invasion, and glycolysis and promote apoptosis of OC cells. Another study found that miRNA-195 (78) could increase Ca^2+^ uptake, reverse glycolysis, and improve OC survival time by inhibiting MICU1 expression in OC. Furthermore, miR-383 (79) can directly target LDHA to inhibit the aerobic glycolysis of OC cells and inhibit cell proliferation and invasion. Notch is another signaling pathway involved in OC cell proliferation, invasion, metastasis, and angiogenesis. Ezh2 (80) is a central target and sensor for glycolytic metabolism in the TME, with multifunctional and anti-apoptotic properties. It can be restricted by the high expression of miRNA101 and miRNA26a, and can directly bind to Notch signaling pathway inhibitors, activate the Notch pathway, stimulate the expression of multifunctional cytokines in T cells and promote their activation, and control effector T cell multifunctionality and survival through Bcl-2 signaling.

**Table 1:** Target sites of ncRNAs in OC glycolysis.

ncRNA	Target	Involvement of other factors	Function	Reference
lncRNA NEAT1	mir-4500	BZW1	Upregulate	(75)
lncRNA 00504	PKM2, HK2, PDK1	miR-1244	Downregulate	(33)
lncRNA 00857	YAP1	miR-486-5p	Upregulate	(18)
lncRNA NRCP	PFKB3		Upregulate	(80)
miRNA101/miRNA26a	EZH2	Bcl-2	Downregulate	(36)
miR-532-3p	MICU1	HIF-1a	Downregulate	(78)
miRNA-195	LDHA	Ca^2+^	Downregulate	(55)
lncRNA HOXB-AS3	LDHA	miR-378a-3p	Downregulate	(76)
circ_0025033	miR-184	LSM4	Downregulate	(73)
lncRNA GEHT1	HIF-1a	LDHA	Upregulate	(31)
lncRNA 00092	PFKB2		Upregulate	(53)
lncRNA AB073614	ERK1/2		Downregulate	(74)
lncRNA oiP5-aS1		Mir-128	Upregulate	(77)
circRNA ITCH		miR-106a	Downregulate	(79)
miRNA-383	LDHA		Downregulate	(80)

## 6 Treatment

Currently, there are various herbal components and chemotherapeutic agents that target glycolytic enzymes or regulatory factors for the treatment of OC. Ginsenoside (20(s)-Rg3) is an antitumor complex extracted from the TCM ginseng (81) and can be used to effectively inhibit glycolysis by inducing the STAT3/HK2 pathway in OC cells. Cardamonin (82) can participate in autophagy in OC cells, inhibit the glycolysis-mediated activation of AMPK, inhibit the activity of HK2 and LDHA, and reduce mTORC1 signaling activation and HK2 expression, which, in turn, reduces the secretion of lactic acid and the production of ATP. The coumarin compound DIC (83) was reported to effectively inhibit pyruvate dehydrogenase kinase (PDK) activity, shift glucose metabolism from glycolysis to OXPHOS, generate higher levels of reactive oxygen species (ROS), attenuate mitochondrial membrane potential, and induce apoptosis. Meanwhile, resveratrol (84) inhibits AKT and mTOR signaling, and decreases glucose uptake and lactic acid production. In addition, resveratrol (85) can activate AMPK and inhibit its phosphorylation, thereby inhibiting the proliferation, migration, and invasion of OC cells and promoting apoptosis of these cells. Baicalein can inhibit the rate of glycolysis by regulating the expression of PGM, HK2, and PDHK1, which are key genes downstream of p53. Heme (86) is an essential cofactor for the electron transport chain and the enzyme ATP synthase in mitochondrial OXPHOS. Enhancement of heme synthesis by exogenous supplementation with the heme precursor 5-aminolevulinic acid reduces HK2 transcription and inhibits glycolysis. The chemotherapy drug isoflurane (87) induces overexpression of HK2, PKM2, and LDHA by promoting the phosphorylation of AKT, leading to an increase in glucose uptake and lactate production, and enhancing the glycolysis rate. A novel PFKB3 inhibitor, 3PO, which enhances the cytotoxic effects of cisplatin on platinum-sensitive and platinum-resistant OC cells, may offer fresh ideas for how to treat OC (88). The PKM2 inhibitor shikonin (89) can inhibit the glycolysis rate, OC growth, and cell migration according to glucose consumption. The Bcl2 inhibitor ABT737 can reverse glycolysis and promote apoptosis through SIRT3-HIF. The receptor tyrosine kinase AXL (90) is thought to be associated with chemoresistance in OC, and studies have confirmed that inhibiting AXL through the phosphorylation of PKM2 not only reduces glucose uptake and lactate production in OC cells, but also enhances the efficacy of cisplatin on OC cells and even reverses cisplatin resistance. Rapamycin (91), which is a macrolide ester produced by *Streptomyces hygroscopicus*, prevents the progression of OC by inhibiting mTOR and blocking the cells in G1 phase. Isopropionolactone (92) effectively targets LDHA, PFK, and HK, glucose consumption and lactic acid production, and is sensitive to cisplatin-induced apoptosis. Thus, targeting glycolytic enzymes, regulatory factors, and signaling pathways may provide another therapeutic approach for OC patients and be a potential therapeutic target.

## 7 Discussion

Glycolysis is a recognized hallmark of tumorigenesis and has provided new therapeutic ideas in oncological research, especially playing a significant therapeutic role in liver cancer and breast cancer. In recent years, a plethora of studies have shown that aberrant expression of glycolytic enzyme activities can serve as a marker of poor prognosis in OC and is strongly associated with low survival. Among them, enhanced expression of three key enzymes in glycolysis—HK, PFK, and PK—can affect OC proliferation, invasion, and metastasis, angiogenesis, and chemoresistance, and can even reverse the glycolytic effect when their activities are inhibited. Glycolytic enzymes also interact with signaling pathways such as AMPK, MAPK, PI3K-AKT, Wnt to promote the malignant process of OC and increase cell proliferation and invasion, metastatic relapse, and chemoresistance of OC, which may be one of the reasons for the low survival rate of OC. In addition, the glycolytic end product lactate can participate in the formation of an immunosuppressive microenvironment, which, in turn, affects chemoresistance and immunosuppression. This is consistent with Bi et al. (15) finding in constructing a prognostic model of OC associated with glycolysis that immune cell infiltration and expression of immune-related genes are related to the TME. HIF-1a can induce a hypoxic microenvironment in OC, and when the expression level of HIF-1a is elevated, it facilitates the cells to make full use of the oxygen when they are hypoxic, confirming that TME favors metabolic reprogramming in cancer. In addition, ncRNAs can regulate glycolytic activity to maintain and promote various malignant behaviors in OC, and some of these ncRNAs can also serve as prognostic markers and potential therapeutic targets for OC. However, for patients with early OC, glycolysis detection, diagnosis, and prognosis assessment are still insufficient. TCM monomers and key enzyme inhibitors may open a new window for the clinical treatment of OC. First, glycolysis activity is directly inhibited by targeting the key enzyme activity; second, it reduces glucose uptake and lactic acid production by inhibiting Glut or LDHA levels; and finally, the level of glycolysis can be indirectly regulated via signaling pathway involved in glycolysis. However, although the application of glycolysis inhibitors has been confirmed, there is no evidence suggesting that glycolysis inhibitors or a combination of classical chemotherapy drugs have been put into clinical use. Therefore, large-scale clinical trials of effective inhibitors and/or combination(s) of classical chemotherapeutic agents are needed. This article only reviews the mechanisms of action of key glycolytic enzymes, transcription factors, ncRNAs and signaling pathways in OC. The chemotherapy resistance, immune escape, and specific regulatory mechanisms still need to be explored in depth.

## Author contributions

CL: Writing – original draft, Writing – review & editing. F-YL: Supervision, Writing – review & editing. YS: Supervision, Writing – review & editing. YT: Writing – review & editing. F-JH: Funding acquisition, Supervision, Writing – review & editing.
